# “Liking” as an early and editable draft of long-run affective value

**DOI:** 10.1371/journal.pbio.3001476

**Published:** 2022-01-05

**Authors:** Peter Dayan

**Affiliations:** 1 MPI for Biological Cybernetics, Tübingen, Germany; 2 University of Tübingen, Tübingen, Germany

## Abstract

Psychological and neural distinctions between the technical concepts of “liking” and “wanting” pose important problems for motivated choice for goods. Why could we “want” something that we do not “like,” or “like” something but be unwilling to exert effort to acquire it? Here, we suggest a framework for answering these questions through the medium of reinforcement learning. We consider “liking” to provide immediate, but preliminary and ultimately cancellable, information about the true, long-run worth of a good. Such initial estimates, viewed through the lens of what is known as potential-based shaping, help solve the temporally complex learning problems faced by animals.

## Abstract 2

What is the distinction between ’liking’ and ’wanting’? Why could we ’want’ something that we do not ’like,’ or ’like’ something but be unwilling to exert effort to acquire it? This Essay argues that the primary hedonic phenomenon called ’liking’ might solve the temporal credit assignment problem for learning that arises when true reinforcement values are available slowly or late.

## Introduction

Berridge and his colleagues [1–4] have long argued that there is a critical difference between “liking” and “wanting.” The scare quotes are copied from papers such as Morales and Berridge’s paper [1] to distinguish the more precise quantities that these authors have in mind from the arguably more blurry everyday meanings of these terms or subjective reports that humans can provide upon verbal request. This distinction has been studied in greatest detail in the case of comestibles such as food and liquid; however, as we will see later, it applies more generally.

Crudely, “liking” concerns the hedonic value of a good such as a food, whereas “wanting” refers to the motivational force that the good can exert in terms of reorganising the behaviour of the agent in its direction (be that by largely Pavlovian mechanisms, as in incentive sensitization [5,6], or also instrumental means [7,8]). “Liking,” which, for comestibles in animals, is typically assessed using characteristic orofacial reactions [9–11], is associated with activity in what is reported as a relatively fragile network of subareas in the gustatory and insular cortex, the ventral striatum, and the ventral pallidum, is broadly unaffected by dopaminergic manipulations but is modulated by opioids. By contrast, “wanting” arises from the robust dopaminergic systems connecting midbrain, striatum, and beyond.

It might seem obvious that, in untechnical terms, liking and wanting should be umbilically connected, so that we like what we want, and vice versa. It is therefore surprising that this is apparently not always to be the case—it is often reported in the context of addiction that drugs that are keenly “wanted” (to a significantly detrimental extent) no longer generate substantial hedonic “liking” [5]. Furthermore, neuroeconomists have delineated an even wider range of utilities [12,13] whose mutual divergence can lead to anomalies. Thus, along with hedonic and decision utility, which are close to “liking” and “wanting,” respectively, are predicted utility (how much the outcome is expected to be “liked”) and remembered utility (what one remembers about how a good was previously “liked”)—and one could imagine “wanting” versions of these latter two utilities also.

The area of food reward casts these issues in rather stark relief [14,15]. Thus, recent evidence is not consistent with the idea that overconsumption and obesity (putatively consequents of over-“wanting”) are increasing because of the devilishly clever “liking”-based hedonic packaging with sweet and fat taste and texture of relatively deleterious foods [16–18]. Instead, careful experiments dissociating the oral sensory experience of foods from their gastric consequences [19–22] suggest that it is the postingestive assessment by the gut of what it receives that is important for the (over)consumption. The substrate of this involving projections via the vagus nerve ending up in the dopamine system [23–25] is quite consistent with a role in “wanting.”

Why then indeed should we have both “liking” and “wanting”? In this essay, we argue that “liking” systems play the role of what is known as potential-based shaping [26] in the context of reinforcement learning (RL; [27]). “Liking” provides a preliminary, editable, draft version of the long-run worth of a good [28]. By providing an early guess at a late true value, this can help with the notorious temporal credit assignment problem in RL [27], which stems from the fact that, in most interesting domains, agents and animals alike have to wait for a long period of time and/or make whole sequences of choices before finding out, much later, whether these were appropriate.

These preliminary, draft, hedonic values thus steer animals towards what will normally be appropriate choices—making learning operate more effectively. RL borrowed the term “shaping” from psychology [29–31] to encompass a number of methods for improving the speed and reliability of learning—just like the effect we are arguing for here. One class of methods systematically adds quantities to “true” underlying rewards; however, like many methods that manipulate utilities, unintended consequences are rife. Potential-based shaping was suggested by Ng and colleagues [26] as a variant that is guaranteed not to have such consequences and indeed is equivalent to a typically optimistic initialization of the estimation of values [32].

In the case of victuals: for survival, animals actually care about the nutritive value of foods (which is why they underpin “wanting”)—this is the long run worth. However, it takes time for the digestive system to process these foods to determine their underlying value, making it difficult to criticise or reward the actions that led to them in the first place. This is exactly the temporal credit assignment problem. Instead, exteroceptive sensory input from the mouth and nose (and even the visual system) underpins a guess at this true value—providing immediate hedonic feedback for the choice of action. Usually, this guess is good, and so the two systems harmonise seamlessly. Given disharmony, it is the nutritive value that should determine ultimate choice, as described above. Thus, even if the orofacial “liking” responses might themselves not be manipulated by “wanting” system substrates such as dopamine, it is by activating dopamine systems in particular patterns that hedonic value can act appropriately.

We first describe conventional model-free methods for prediction in RL, and the role of potential-based shaping in this. We then use the case of flavour–nutrient conditioning to suggest how the systems concerned might interact. Finally, in the discussion, we touch on some more speculative suggestions about the underlying source of utility in the context of homeostatic RL [33] and discuss a version of the same argument, but now for aesthetic value [34].

### Model-free RL

In the main part of this essay, we concentrate on Pavlovian conditioning [35]—the case in which predictions about future, potentially valuable outcomes lead to automatic actions such as approach, engagement, and even licking (whether or not those actions are actually useful for acquiring those outcomes; [36]). Thus, we focus on problems of evaluation and save consideration of the choice between actions for later.

We consider a Markov prediction problem in a terminating, episodic case with no temporal discounting. Here, there are connected, nonterminal states, s∈S, a special terminating state s*, a transition matrix among just the nonterminal states,

$T_{ss^{\prime }}=P(s_{t+1}=s^{\prime }|s_{t}=s)$, $\{s,s^{\prime }\}\in S$, with the remaining probability being assigned to the terminating state ${\tilde{T}}_{s}=1-\sum_{s^{\prime }\in S}T_{ss^{\prime }}=P(s_{t+1}=s^{*}|s_{t}=s)$ and rewards $r_{s}\in R$ associated with state *s* (which we will assume to be deterministic for convenience; also writing vector **r** for the rewards for all states); and *r*_s*_ = 0.

Then, if we write $V_{s}=E_{s_{1}=s}[\sum_{t=1}^{\infty }r_{s_{t}}]$ for the long run value of state s∈S (the value of s* is 0), and vector **V** for all the values, we have

$$V_{s}=r_{s}+\sum_{s^{\prime }\in S}T_{ss^{\prime }}V_{s^{\prime }}\;\mathrm{or}$$ (1)

$$V=r+TV={\left[I-T\right]}^{-1}r$$ (2)

by writing the recursion directly (and noting that T excludes the terminating state, which means that I−T is invertible).

The simplest form of temporal difference (TD) learning [27,37] attempts to learn the values *V*_*s*_ from stochastic trajectories *s*_1_, *s*_2_, *s*_3_,…,s* generated by sampling from T. TD accomplishes this by constructing a prediction error from the sampled difference between right and left side of Eq 1

$$\delta_{t}=r_{s_{t}}+V_{s_{t+1}}-V_{s_{t}}$$ (3)

and applying

$$V_{s_{t}}=V_{s_{t}}+\alpha \delta_{t}$$ (4)

where *α* is the learning rate. There is substantial evidence that the phasic activity of at least some dopamine neurons in the ventral tegmental area (VTA) of the midbrain, and the release of dopamine in target regions such as the nucleus accumbens, reports the TD prediction error *δ*_*t*_ of Eq 3 [7,38–42].

In cases of instrumental conditioning, when actions must also be chosen, the prediction error *δ*_*t*_ can also be used to critize a choice (in an architecture called the actor-critic; [43]). The idea is that actions that lead to either unexpected good rewards (judged by $r_{s_{t}}$) or unexpectedly good states (judged by large predicted long-run future rewards, $V_{s_{t+1}}$) should be more likely to be chosen in the future. This can be measured by *δ*_*t*_.

**Fig 1:**
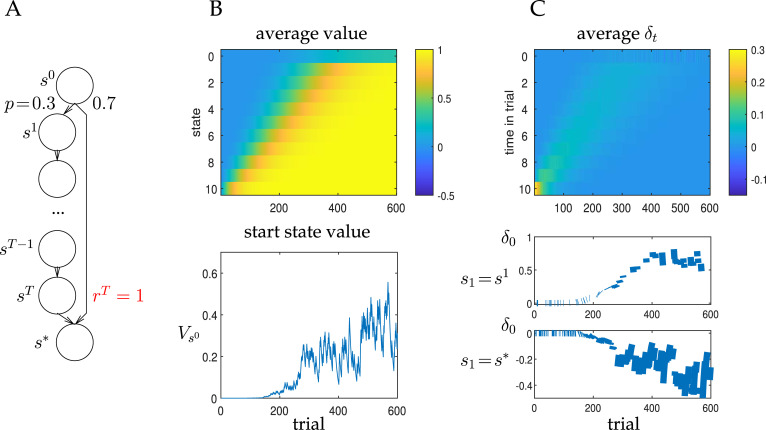
TD-based Markov prediction. (A) Simple Markov prediction problem with a tasty morsel provided at *t* = 1 (*s* = *s*^1^) with probability *p* = 0.3, which leads to a digestive reward of *r*^*T*^ = 1 at time *T*. (B) Evolution of the value for the application of TD learning to the case that *T* = 10. Upper plot: average over 1,000 simulations (here, and in later figures, we label state *s*^*i*^ by just its index *i*); lower plot: single simulation showing $V_{s^{0}}$. (C) Evolution of the TD prediction error *δ*_*t*_ over the same trials. Upper plot: average over 1,000 simulations; lower plots: single simulation showing *δ*_0_ for a transition to *s* = *s*^1^ (above); or to *s* = s* (below). Here, *α* = 0.1. TD, temporal difference.

Although TD learning is powerful, offering various guarantees of convergence when the learning rate *α* satifies suitable conditions, it has the problem of being sometimes slow. To illustrate this, we consider a case related to the one that we will consider later in flavour–nutrient conditioning. Fig 1A shows a case in which from a start state *s* = *s*^0^, there is a high probability (*p* = 0.7) transition directly to the terminal state s*, and a low probability transition to state *s* = *s*^1^, associated with an observation (later modelling the oral sensation of a morsel of food) and which initiates a sequence of *T* states leading to a rewarding outcome *r*^*T*^ = 1 (later modelling the gut’s evaluation of this morsel) and then the terminal state s*. Fig 1B depicts the course of learning of the value structure associated with selected states, applying Eqs 3 and 4. The upper plot depicts the average value (across 1,000 simulations) for all nonterminal states as a function of learning trial. As expected for this sort of complete serial compound stimulus representation [44,45] in which every time step following the morsel of food is separately individuated, the value of the reward available at *s*^*T*^ apparently propagates backwards to *s*^1^. The further propagation to *s*^0^ is then affected by the stochasticity at that single state. The lower plot shows the evolution of $V_{s^{0}}$ for one single run; the slow rise and stochastic fluctuations are evident.

Fig 1C shows the prediction errors that occasion the learning of the values shown in Fig 1B. For convenience, in the single example beneath, we have separated the case that the transition from *s*^0^ is to *s*^1^, and ultimately to the actual reward at *s*^*T*^ (upper) from the case that the transition is to s*, and thus no reward (lower). Given that the average value of $V_{s^{0}}=p=0.3$, the former transition is associated with a positive prediction error; the latter with a negative one. Note that at the end of learning, the only prediction error arises at time *t* = 0, because of the stochasticity associated with the transition to *s*^1^ versus s*. At all other states, predictions are deterministically correct. Again, with the complete serial compound stimulus representation, over the course of learning, the prediction error apparently moves backwards in time during the trial—a phenomenon that has been empirically rather elusive, at least until very recently [46].

The most salient characteristic of the learning in this case is its sloth—apparent in the averages and the single instance. There are two reasons for this: First, *p* is low, which means that the agent usually fails to sample *s*^1^ and the cascade leading to the reward. The second is that the learning rate *α* = 0.1 is rather modest. Increasing *α* leads to faster learning, but also greater fluctuations in the values and prediction errors. Particularly, in this simple case, it would be possible to speed up learning somewhat by using a temporally extended representation of the stimulus [45,47] or an eligibility trace (the λ in TD(λ); [37]). However, in general circumstances, these can be associated with substantial variability or noise—particularly for long gaps as between ingestion and digestion—and so would not be a panacea in our case. Sophisticated modern models of conditioning that provide a substantially more neurobiologically faithful model of the learning in temporally extended cases (e.g., [48]) also currently concentrate on relatively modest time gaps.

### Potential-based shaping

Shaping was originally suggested in the context of policy learning as a way of leading subjects through a sequence of steps in order to facilitate learning of good performance in a particular task [30]. The idea is to provide a set of intermediate (typically state- and/or action-dependent) rewards that are different from those specified by the task itself in order to provide an easier path for animals to learn appropriate final behaviour. The benefit of this has also been recognised in RL (e.g., [26,49], also leading to ideas about intrinsic rewards [50], by contrast with the extrinsic rewards that are determined by the task). The benefits of such intermediate rewards come on top of those of improved representations such as those mentioned above.

Citing entertaining examples such as the microcircling bicycle of Randløv and Alstrøm [49], Ng and colleagues [26] observed that manipulating the reward structure (*r*_*s*_ in our terms) can have unintended consequences—skewing predictions (and, in instrumental cases, choices) away from their optimal values for the original task. They therefore suggested a scheme called potential-based shaping, which could steer learning but with a guarantee of no asymptotic effect. This involves adding a function of state *ϕ*_*s*_ to TD error terms such as that in Eq 3, making it

$$\delta_{t}=r_{s_{t}}+[\phi_{s_{t+1}}-\phi_{s_{t}}]+V_{s_{t+1}}-V_{s_{t}}.$$ (5)

The name potential-based shaping comes from the fact that summing the net effect of *ϕ* in cycles of states is 0, because it appears in difference form—thus, it satisfies the same sort of no-curl condition as a conventional potential function. This means that it does not distort the values ascribed to states at the asymptote of learning when the predictions have converged. However, the idea is that the shaping function provides a hint about the values of states—being large for states that are associated with large long-run reward. Thus, a transition from a state *s*_*t*_ = *s* to *s*_*t*+1_ = *s*′ when *ϕ*_*s*_ is low and *ϕ*_*s*′_ is high will provide immediate positive error information allowing the value *V*_*s*_ for state *s* to be increased even if *V*_*s*′_ has not yet been learned and so is still 0. In an instrumental conditioning case, the resulting high value of *δ*_*t*_ will also be useful information that the action just taken that led to this reward, and transition is also unexpectedly good and so is worth favouring (as a form of conditioned reinforcement; [51]).

For the Markov prediction problem of Fig 1, the appropriate shaping function associated with the morsel of food is rather straightforward—it should be *ϕ*_*s*_ = 1 for *s* = *s*^1^…*s*^*T*−1^ and $\phi_{s^{T}}=0$. The reason is that ingestion of the morsel with its sweet taste (at *s*^1^) predicts the benefit of digestion (at *s*^*T*^) for all those future times. Formally, the hedonic value is generated by $\phi_{s_{t+1}}-\phi_{s_{t}}$.

**Fig 2:**
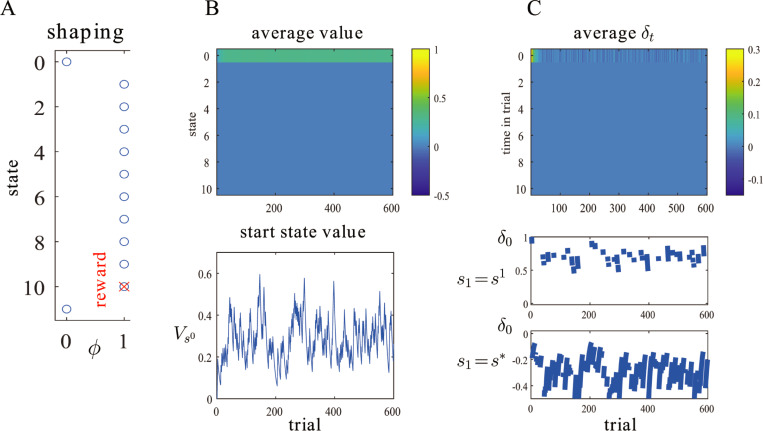
TD-based Markov prediction with perfect shaping. (A) The ideal shaping function *ϕ* (blue circles) is 1 after acquisition of the food (at *s*^1^) until the reward arrives (red cross at *s*^*T*^). (B) Evolution of the value for the application of TD learning to the case that *T* = 10. Upper plot: average over 1,000 simulations; lower plot: single simulation showing $V_{s^{0}}$. (C) Evolution of the TD prediction error *δ*_*t*_ over the same trials. Upper plot: average over 1,000 simulations; lower plots: single simulation showing *δ*_0_ for a transition to *s* = *s*^1^ (above); or to *s* = s* (below). Here, *α* = 0.1. TD, temporal difference.

Fig 2 shows the course of learning in the Markov prediction problem of Fig 1, given these perfect shaping function (shown in Fig 2A). It is apparent that acquisition of the correct value for $V_{s^{0}}$ is greatly accelerated, as is the advent of the correct set of prediction errors (which are immediately zero for *s*≠*s*^0^). This shows the benefit of shaping. The agent can learn quickly that the state giving access to the morsel of food is itself appetitive. Furthermore, in a more complex problem in which there is a choice between actions, one of which provided access to *s*^0^, this action could also be learned as being worth 0.3 units of reward.

Note also an important difference between Figs 1B and 2B—namely that, at the end of learning, *V*_*s*_ = 0 for *s* = *s*^*τ*^, *τ*≥1 in the latter, but not the former. The reason for this is that the prediction error is 0 for *t*≠0 because of the perfection of the shaping function—implying that there is nothing to learn for the states that lie between ingestion and digestion. Thus, Fig 2C shows that there is no prediction error within a trial either (and so backward propagation thereof), except just at the start state. In fact, the total prediction of the long-run reward from a state is *V*_*s*_+*ϕ*_*s*_. It has thus also been observed that a perfect substitute for this sort of potential-based shaping is to initialize *V*_*s*_ = *ϕ*_*s*_, and then use standard TD learning, as in Eqs 3 and 4 [32]. However, although this is technically correct, it is not suitable for our purposes of flavour–nutrient conditioning since it does not respect a separation between taste processing and conditioning mechanisms.

**Fig 3:**
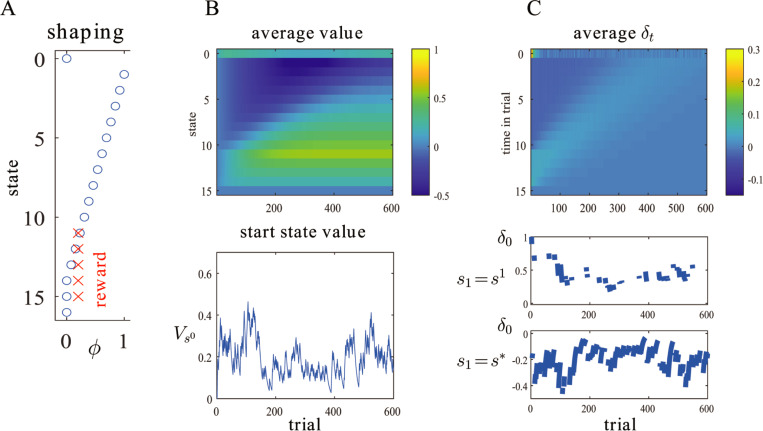
TD-based Markov prediction with a partial shaping function. (A) A suboptimal shaping function *ϕ* that decreases from 1 to 0 linearly after acquisition of the food (at *s*^1^), and with reward spread over five time steps (red crosses; note the extension of the state space to *T* = 15). (B) Evolution of the value for the application of TD learning to this case. Upper plot: average over 1,000 simulations; lower plot: single simulation showing $V_{s^{0}}$. (C) Evolution of the TD prediction error *δ*_*t*_ over the same trials. Upper plot: average over 1,000 simulations; lower plots: single simulation showing *δ*_0_ for a transition to *s* = *s*^1^ (above); or to *s* = s* (below). Here, *α* = 0.1. TD, temporal difference.

If the shaping function *ϕ*_*s*_ is not perfect, then the course of learning will be at least partially disrupted. Fig 3 shows a case in which the shaping function decays linearly from $\phi_{s^{1}}=1$, as if the prediction from the taste system associated with the future digestive benefit cannot last as long as the time that the gut takes to process the food morsel. Furthermore, as a very abstract model of the time the digestive system might take to process the food, the same total reward is spread over five time steps.

In this case, the prediction $V_{s^{0}}$ learns very quickly at first, but then temporarily modestly decreases (between around trials 200 to 400 in the example) before recovering. The suppression arises since *δ*_*t*_<0 for *t* = 1…*T*−1 on early learning trials (since $\phi_{s^{t}}$ is decaying linearly over these times), and this negative prediction error propagates backwards to influence *V*_0_. Later, the positive prediction error that starts associated with the digestive report of the nutritive value (i.e., *r*^*T*^ = 1) itself propagates back to overwhelm the suppression. Furthermore, the asymptotic value *V*_*s*_ comes over the course of learning exactly to compensate for the inadequacy of the shaping function such that *V*_*s*_+*ϕ*_*s*_ is the long-run reward from state *s*.

### Flavour–nutrient conditioning

Flavour–nutrient conditioning has a venerable history [14,15,19,52–55]. The idea is to separate the impact of any immediate sensory input associated with a liquid or food: taste, smell, sight, oral texture, whisker responses, and the like from what is sometimes known as its postoral effects—the results of processing in the stomach, gut and beyond. The key questions are which of these drives “liking” and “wanting” for the consumable.

One of the most popular methods is to use a form of so-called electronic oesophagus [19]. With this, an animal can be allowed to sample substances orally by licking them, but the licks are paired with the delivery of a potentially different substance directly into the stomach of an animal through a catheter. Thus, it is possible to dissociate fully the various sensory qualities of an ingestible substance from its digestible nutrient content and to assess issues such as an animal’s ability to learn about the relationship between an originally neutral flavour and appetitive or aversive digestive consequences.

The result of an extensive body of work in this direction is quite consistent with the separation between “liking” and “wanting” [1]. The immediate hedonic quality of consumables, associated with “liking,” is assessed rapidly by exteroceptive sensory systems based on connections to primary and sensory taste cortex, amygdala, insular cortex, and beyond. The influence of consumables on long-run consumption (and motivational attraction), associated with “wanting,” is assessed more slowly by interoceptive mechanisms, with ultimate connections via the vagus nerve (and possibly dorsal root ganglia) to the dopamine system [23–25].

**Fig 4:**
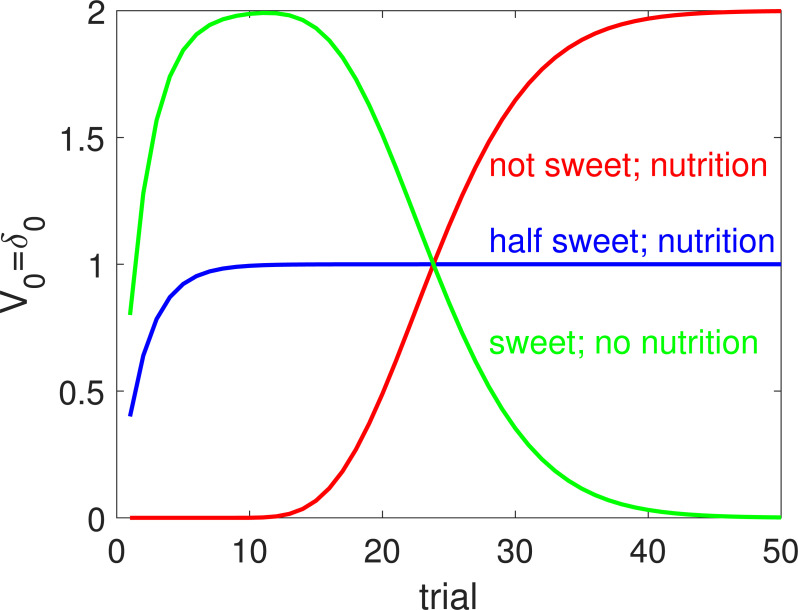
Simulation of flavour–nutrient conditioning. The lines show the evolution of the learned value of three different flavours with orthogonalized intrinsic sweetness and nutritive values. The red flavour is not sweet but is highly nutritious—and so lacks shaping (as in Fig 1). The green flavour is very sweet (with a shaping function reflecting this) but is not nutritious. The blue flavour is somewhat sweet and somewhat nutritious and is also associated with a perfect shaping function (as in Fig 2). Here, transitions are deterministic and *α* = 0.4.

In order to illustrate the effect of paradigms in which exteroceptive and interoceptive qualities are orthogonalized, we simulated a version of the shaping paradigm described in the previous section, but with foods of three separate flavours associated with three different nutritive values. One (shown by the red line in Fig 4) is not at all sweet, and so lacks any shaping reward, but is highly nutritious (thus, slightly abusing notion: *ϕ*^red^ = 0; *r*^red^ = 2). A second (green) is very sweet, attracting substantial shaping, but lacks any nutritive value (*ϕ*^green^ = 2; *r*^green^ = 0). The final flavour (blue) is of intermediate sweetness and nutrition (*ϕ*^blue^ = 1; *r*^blue^ = 1)—but in such a way that these two qualities match (at least given the prevailing motivational state; [56]). Here, for convenience, we consider a deterministic case in which each flavour is treated separately, and with a faster learning rate than in the previous section (*α* = 0.4).

Fig 4 shows the course of learning of the value that is assigned to each of the three flavours over the course of exposures. To our knowledge, this particular experiment has not been performed, so these quantities could be seen as predictions of relative preference in an appropriate test of choice. The purely nutritive, nonsweet flavour (red) only gains value slowly but ultimately reaches a high asymptote. Learning is delayed without the benefit of shaping. The purely sweet, nonnutritive flavour (green) becomes attractive very rapidly, because it outwits the shaping mechanism. However, ultimately, the nutritive value dominates, and so its ultimate value reduces to 0. Finally, the conventional, modestly appetitive flavour (blue) shows the fast time course of learning evident also in the previous section, since the shaping function is correct—with “liking” and “wanting” being aligned.

## Discussion

In this essay, we provided an RL view of “liking” and “wanting,” which uses the construct of potential-based shaping [26] as a basis for a hedonic signal inspired by a sensory object. This steers an RL rule such as TD learning when veridical information about the long-run worth of that object may arise only slowly. It does so by providing standard RL methods of learning the relationship between conditioned stimuli (e.g., the sensory qualities of the food) and the digestive equivalent of unconditioned stimuli (the true worth) with a substantial head start.

We illustrated this argument using modern conceptions of flavour–nutrient conditioning, because some of the most extensive data and discussions on the distinction between “liking” and “wanting” have arisen in this domain. Here, “liking” provides a preliminary assessment of the long-run worth of a morsel of food or a drop of liquid. The latter is ultimately reported by postoral evaluation mechanisms feeding into the dopamine system and is the substrate for establishing the motivational impact or “wanting” for those foodstuffs. Our simulation was extremely simplified—e.g., with deterministic timing between “liking” and “wanting” signals that do not capture anything of the complexities of gastric dynamics and gastric emptying or the like. With more elaborate experimental paradigms, it will be possible to constrain more faithful models and use causal manipulations to test them.

The requirements on “liking” to be perfectly aligned with “wanting” are relatively stringent. One necessity is for an assessment of the long-run value to be made based on the rather remote information provided through oral evaluation. This is particularly hard in the era of processed foods (and artificial sweeteners, e.g., [57]), as methods for making prestidigitators out of taste and olfactory receptors abound. One prominent anomaly is that fructose, which can actually be sweeter to the taste than glucose at equivalent concentrations and is a key raw material for lipogenesis in the liver (thus having advantageous digestive import), apparently fails to generate substantial postoral “wanting” signals [58]. The full range of learning anomalies to which this leads has yet to be mined. Furthermore, there are various experimental procedures that can make persistently dis-“liked” goods strongly “wanted” [59,60].

An important and controversial set of subtleties on which the literature does not seem completely settled is the precise roles of dopamine in these processes [61–64]. This is important because of dopamine’s multifaceted role in appetitive learning—including representing the sort of phasic TD prediction error for reward that we wrote in Eq 3 [7,38] and a more tonic representation of the reward availability in an environment [65,66]. One source of complexity is the potentially differential role of various parts and projections of the dopamine system—notably the VTA and the substantia nigra pars compacta (SNc), connected, respectively, with the ventral and dorsal striatum and often implicated, respectively (though perhaps incompletely; [67]), in value learning (and Pavlovian responses) and action learning [7,8].

There is excellent evidence that dopamine is not involved in the main orofacial reactions that are taken as the ground truth for “liking,” but that it is deeply implicated in “wanting” [64,68]. However, nonnutritive but orally attractive sweeteners such as sucralose do lead to the activity of dopamine neurons (e.g., [23]), and Tellez and colleagues [25] reported that the release of dopamine into the ventral (but not dorsal) striatum was identical for sucralose and sucrose. This would be consistent with the potential-based shaping that we have described. Nevertheless, while Han and colleagues [24,25] reported a separation between hedonic and nutritive aspects of sucrose, with a critical role only for dopamine in the dorsal striatum (and a pathway to this associated with neurons in the right nodose ganglion of the right side of the vagus nerve), Fernandes and colleagues [23] reported that the postingestive import of sucrose (relative to sucralose) is mediated, at least in substantial part, by connections running via the left side of the vagus nerve (the left nodose ganglion) to VTA dopamine neurons (and thus presumably the ventral striatum), and that it is activation of this pathway that can sustain vigorous operant behaviour.

These subtleties mainly concern the alignment of “liking” and “wanting” in terms of value. The other aspect of alignment, highlighted by Fig 3 concerns timing. An optimal shaping function would remain high for the whole length of time until the report of the “true” worth of a sensory object is available. This is hard by itself; and maintaining information about which object inspired which later signal of true worth would seem impossible. Indeed, paradigms in which nutritive and nonnutritive pellets of food are provided less than two minutes apart show that the ascription of pellet to consequence can be rendered highly imperfect [69]. It would be interesting to examine whether hedonic systems can sustain relatively more tonic activity, noting that, under the shaping hypothesis, this might not be apparent in the activity of dopamine neurons (since, as evident in Fig 1, the prediction error becomes 0).

One wider context for this work is a progressive blurring within RL of the understanding of utility and reward as being defined by the environment versus the animal or agent itself [33,70–72]. A prominent example of this comes from the field of homeostatic RL [33] and was also explicitly tied to flavour–nutrient conditioning. This theory starts from the oddly frequently overlooked point that the environment does not provide any external evaluation even of primary reinforcers (such as food or liquid). Instead, through reproductive sorting, evolution has presumably programmed a set of internal mechanisms for evaluating primary reinforcers that have been found, historically, to benefit fitness. Keramati and Gutkin [33] formulated this problem via the notion of an optimal homeostatic set point for various physiologically important variables in a complex internal state space plus the suggestion that internal utility is generated by movement relative to this set point. In a form of generalised drive reduction theory [73], movement towards the set point (reducing the drive associated with the aspects of the state that were dysregulated) would be appetitive, associated with positive reward *r*; movement away would be aversive, associated with negative reward *r*.

The potential-based shaping version of this [28] suggests that, indeed, there is no external reward at all *r*_*t*_ = 0,∀*t*. However, instead, evolution has endowed us with a large-scale shaping function that nominally estimates a scalar quantity of external semantics and significance, such as expected lifetime—from information about internal state (of nutrition, hydration, and the like) [74]. Then changes in internal state that increase or decrease lifetime generate positive or negative contributions, respectively, to the prediction error of Eq 5 and substitute for external reward. Alternatively, we could have been endowed directly with what amounts to the derivative of this function $\phi_{s_{t+1}}-\phi_{s_{t}}$, which is the only way that the shaping function appears in practice.

In terms of the argument in our essay, there could be both cortical (“high road”; putatively involving areas such as the insular cortex) and subcortical (“low road”) such shaping functions or derivatives, which respond to physiological signals [14]. Thus, we would generalise from a hedonic-based shaping function (from exteroceptive sensation) coupled to a ground-truth reward function associated with nutritive properties to the sum of two different shaping functions—a hedonic, “liking”-associated, exteroceptive one and a ground-truth, “wanting”-associated, interoceptive one. One can imagine further generalising such a scheme.

A second example comes from a recent theory for the basis of aesthetic value for sensory objects such as pictures [34]. The idea is that an animal should ideally have a sensory system that is efficient at performing information processing on the distribution of sensory inputs that it is likely to experience in the future. This efficiency is commonly defined in the Helmholtzian terms of the fit of an internal generative model to this distribution [75], implying that a suitable shaping function based on the state of the sensory processing system might be a measure of this fit (the Kullback–Liebler divergence, or average log probability, in [34]).

Consider the case that hedonic “liking” is generated by the change in the value of this shaping function consequent on observing a sensory input. Where would a change to the average log probability of likely future stimuli come from? Brielmann and Dayan [34] suggested that making such an observation has two relevant effects. One is a direct form of plasticity: changing the state of the sensory system so that it awards a higher likelihood to that particular input. The worth of this change is exactly what one large class of theories considers to generate aesthetic value for stimuli—these are the theories that concentrate on learning progress or prediction error [76]. A second natural consequence of observing an input is to expect that this input is at least a little more likely to arise in the future [77]. The worth of this change turns out to be closely related to the efficiency with which the input can currently be processed (assuming that the plasticity referred to above is modest). This notion of efficiency is the basis of a second, and traditionally competing, popular class of theories for aesthetic value [78–80]. Thus a potential-based shaping theory of “liking” unifies these two concepts of aesthetic value. How or whether the equivalent of “wanting” is calculated or represented is less clear.

Throughout our simulations, we assumed that the shaping function *ϕ*_*s*_ was fixed. However, in fact, there is ample evidence for what is known as hedonic shift learning—e.g., the well-known Garcia effect, that pairing food with subsequent sickness (including gastric malaise) has a powerful impact on creating dis-“liking” (even when the particular food itself was not the pathological agent) [81]. From a formal viewpoint, provided that changes to *ϕ*_*s*_ are not happening continually throughout the course of the sort of RL that we have covered, such changes would not disturb the asymptotic net values (because *ϕ*_*s*_ is used as a potential function). However, such changes can certainly change the speed of learning, as we have shown. Furthermore, although we have not discussed it here, since we considered value rather than action learning, it could influence the willingness of animals to explore the food sufficiently to find out that it was actually not responsible for the malaise. Such path dependencies have been suggested as being important contributors to other aspects of maladaptive behaviour [82].

It is therefore of great interest to understand the psychological and neural rules governing hedonic shift learning. However, an original expectation that advantageous or disadvantageous interoceptive discoveries about the nutritive quality of foods or liquids with novel tastes would exert their entire effect by increasing or decreasing the hedonic pleasure of those tastes does not seem to have been borne out [53]. Indeed, the extent of this latter change pales in comparison with one associated with what is a separate and powerful form of “wanting.” This asymmetry is perhaps in keeping with the reported fragility of the “liking” system [5].

Based originally on some gustatory Italian misadventures, Dickinson and Balleine [83] suggested that hedonic shift learning was a way that a goal-directed instrumental control system could be instructed about the “true,” bodily value of an affectively charged outcome—a phenomenon they called incentive learning. In RL terms, this would be a way by which a model-based system [84,85] could help decide which goals are potentially worth pursuing. Since, as we noted, hedonic shift learning is incomplete, this form of incentive learning would suffer limits.

The form of involvement of the dopamine system in “wanting” is rather suggestive of model-free control. However, the paradigms we have discussed do not provide clear evidence about the extent to which when “wanting” separates from “liking,” the “wanting” value can influence model-based control, as it normatively should.

In sum, we have provided an account of “liking” in terms of an RL theory of potential-based shaping. We used the example of flavour–nutrient conditioning to show how “liking” could be aligned with “wanting” and to show some of its desirable properties in terms of speeding learning when this happens. We also noted links with homeostatic RL, where multiple layers of extero- and interoceptive shaping might be combined, and to the hedonics associated with sensory aesthetics. “Liking” amounts to a loan that, provided it is paid back in a timely manner by processes associated with “wanting,” will organise the smooth coordination of learning and behaviour.

## Abbreviations

RL: reinforcement learning
SNc: substantia nigra pars compacta
TD: temporal difference
VTA: ventral tegmental area
